# Editorial: Bacteriophages in the fight against foodborne pathogens

**DOI:** 10.3389/fmicb.2023.1269824

**Published:** 2023-08-22

**Authors:** Ana Oliveira, Alexandre Lamas, Silvio B. Santos

**Affiliations:** ^1^Centre of Biological Engineering, University of Minho, Braga, Portugal; ^2^LABBELS—Associate Laboratory, Braga, Portugal; ^3^Food Hygiene, Inspection and Control Laboratory (LHICA), Department of Analytical Chemistry, Nutrition and Bromatology, Veterinary School, Campus Terra, Universidade da Santiago de Compostela, Lugo, Spain

**Keywords:** bacteriophage, *Salmonella*, *Escherichia coli*, *Aeromonas*, *Vibrio*, food safety

Foodborne pathogens represent one of the main public health problems worldwide. The emergence of multidrug-resistant strains significantly increases the risk of their caused infections for both livestock and consumers. As conventional antimicrobials, biocides, and preservatives prove insufficient, scientists have rekindled their relationship with an old ally forgotten for decades: the bacteriophages. These viruses, known for their specific targeting capabilities against bacterial species, serotypes, and strains, offer a promising avenue for developing targeted and effective antimicrobial solutions in the food production chain. These viruses are natural predators of bacteria characterized by their specificity, which can go up to the strain level, opening a promising avenue for developing targeted and effective antimicrobial solutions in the food production chain. Phage potential is not restricted to a particular point of the food production chain. They can be applied for treating animals on farms, for the disinfection of food surfaces, or even added directly to food or food packaging. However, to make the use of phages in the food chain a reality, applicability studies are needed. This Research Topic showcases six groundbreaking studies that exemplify the potential of bacteriophages in ensuring food safety and combating foodborne pathogens, including isolation, genotypic and phenotypic characterization, and the practical application of bacteriophages against different foodborne pathogens.

One of the challenges of treating intensively raised chickens, with thousands of animals per farm, is the administration route. The inclusion of phages in drinking water or feed presents a promising alternative. Focusing on primary production, Kuźmińska-Bajor et al. evaluated the UPWr-S134 bacteriophage cocktail composed of three *Jerseyvirus* bacteriophages to treat broiler chickens infected with *Salmonella* Enteritidis. The authors of this study evaluated the stability of this cocktail in simulated gastric fluid (SGF) and observed that while the SGF inactivated phages when administered alone, the presence of feed prevented this inactivation. It was demonstrated that feed is a promising formula for administering phages. Moreover, the results indicated that the oral administration of phages in *S*. Enteritidis-infected chickens managed to reduce the levels of this pathogen in the internal organs of the animals.

Representing the next stage of the food chain, Al-Hindi et al. evaluated the application of the lytic *Jerseyvirus* bacteriophage LPSent1 for the control of *S*. Enteritidis multi-resistant strains in food. The phage demonstrated the ability to reduce bacterial contamination in milk, apple juice, and chicken breast foods by up to 5 log_10_ CFU/g, both at 25°C and refrigeration temperature, with some ability to reduce biofilm formation. However, the challenge lies in the broad lytic spectrum needed to tackle the more than 2,600 different serotypes of *Salmonella*, something that can be circumvented through the use of phage cocktails with a broad or complimentary lytic spectrum or even the design of genetically modified phages able to achieve such spectrum.

Enterohemorrhagic *Escherichia coli* (EHEC) and enterotoxigenic *E. coli* (ETEC) are foodborne pathogens responsible for major outbreaks worldwide. Zhou et al. evaluated the ability of the *Mosigvirus* lytic bacteriophage vB_EcoM_SQ17 to control EHEC and ETEC O157:H7 in milk, beef, and lettuce. This phage demonstrated a high capacity in reducing *E. coli* contamination levels at refrigeration temperatures in these three matrices.

Hou et al. evaluated the jumbo phage ZPAH34 against a multi-resistant strain of *Aeromonas hydrophila*, an emerging pathogen. Curiously, despite being a jumbo phage, it showed the smallest dimension reported to date in a phage with these characteristics. The ZPAH34 phage effectively inhibited biofilm formation and partially eradicated preformed biofilms. Application of ZPAH34 in fish filets and lettuce significantly reduced pathogen levels, offering promising prospects for combating emerging pathogens in aquatic food products.

Gao et al. isolated and characterized the *Maculvirus* lytic phage OY1 against *Vibrio* spp. The phage demonstrated a broad lytic spectrum and the ability to both inhibit biofilm formation and destroy preformed biofilms as well as to reduce *Vibrio* spp. in fish muscle extract juice. Considering the increasing importance of aquaculture in the global food supply, the negative impact caused by *Vibrio* spp., and the existence of a depuration process in seafood before being marketed, the application of phages in depuration water represents an interesting and viable approach to minimize pathogenic bacteria transmission.

Finally, Sukjoi et al. evaluated the therapeutic effects of oral administration of bacteriophages ST-W77 and SEW109 to treat non-typhoid salmonellosis using mice and *S*. Typhimurium as a model host and a strain, respectively. Oral administration of these phages reduced bacterial invasion and inflammatory response in colonic epithelial cells while maintaining phages in the intestinal lumen, showcasing their therapeutic potential in combating *Salmonella* Typhimurium infections.

While the studies presented in this Research Topic demonstrate the enormous potential of phages to control foodborne pathogens in the food production chain, several challenges remain. Determining the optimal forms of application, managing bacterial resistance, and exploring phage proteins as alternatives to whole phages are critical areas of focus. The journey ahead may be filled with obstacles, but the prospect of revolutionizing food safety through bacteriophages promises an exciting and transformative path for the scientific community.

## Author contributions

AO: Writing—review and editing. AL: Writing—original draft. SBS: Writing—review and editing.

